# Antimicrobial resistance of *Escherichia coli* and *Enterococcus* spp. isolated from Estonian cattle and swine from 2010 to 2015

**DOI:** 10.1186/s13028-019-0441-9

**Published:** 2019-01-21

**Authors:** Birgit Aasmäe, Liidia Häkkinen, Tanel Kaart, Piret Kalmus

**Affiliations:** 1 Estonian University of Life Sciences, Kreutzwaldi 62, Tartu, Estonia; 2 Department of Bacteriology-Pathology, Estonian Veterinary and Food Laboratory, Kreutzwaldi 30, Tartu, Estonia

**Keywords:** Antimicrobial resistance, Cattle, *E. coli*, Enterococci, Swine

## Abstract

**Background:**

The prevalence of resistant *Escherichia coli* and *Enterococcus* spp. in food-producing animals has increased worldwide. The objective of the study was to investigate the occurrence of antimicrobial resistance of *Escherichia coli, Enterococcus faecium* and *Enterococcus faecalis* isolated from healthy and diseased swine and cattle in Estonia. Clinical specimen and faecal samples were collected during 2010 to 2015. The in vitro antimicrobial susceptibility was determined using the microdilution method.

**Results:**

The most prevalent resistance of *E. coli* isolates from clinically healthy swine was observed against streptomycin (39.2%), tetracycline (32.5%) and sulfamethoxazole (30.0%), whereas in clinically healthy cattle, the resistance was the highest against aminoglycosides (7.0–8.8%) and tetracycline (7.0%). The *E. coli* isolates from clinically healthy swine showed significantly higher multidrug-resistance compared to isolates originated from clinically healthy cattle. *E. coli* isolates from diseased swine showed highest resistance to sulfamethoxazole (68.6%), tetracycline (60.2%) and streptomycin (54.6%). The proportion of resistant *E. coli* isolates from diseased cattle (clinical submissions) was highest to streptomycin (63.5%), sulfamethoxazole (60.3%) and tetracycline (58.8%). The proportion of multidrug-resistant isolates did not differ significantly between animal species. Among *E. coli* isolates, four strains representing AmpC phenotypes were found. One plasmid-encoded AmpC type β-lactamases producing *E. coli* from clinically healthy cattle was found to harbour the *bla*_CMY-1_ gene, and another from clinically healthy swine carried the *bla*_CMY-2_ gene. Among nine *E. coli* strains exhibiting an ESBL phenotype three strains was found to be the same genotype *bla*_TEM-52C_. Enterococci from healthy swine and cattle showed high resistance to tetracycline and erythromycin. Regarding enterococci, the number of multidrug-resistant strains was significantly higher in swine isolates compared to isolates originated from cattle.

**Conclusions:**

The antimicrobial resistance of *E. coli* isolates was high in both Estonian swine and cattle. However, swine isolates, especially *E. coli* from healthy swine, had developed a higher level of resistance. The amount of multidrug-resistant *E. coli* isolates was also significantly higher in clinically healthy swine compared to that in cattle.

## Background

Bacterial infections are one of the most prevalent groups of diseases in production animals and are commonly treated with antimicrobial drugs. Antibacterial treatment is essential to treat diseased animals; however, one of the negative impacts is expansion of antimicrobial resistance. The antimicrobial resistance of bacterial species originating from production animals also influences human health through the transfer of resistant organisms or genes via food chain [1, 2]. The extended spectrum beta-lactamase (ESBL)-positive *Escherichia coli* isolates in food-producing animals have been frequently identified [3]. As the AmpC and ESBL producing strains are detected in cattle and swine, there is a potential risk for transmission of the strains to other animals and humans [1].

Intestinal commensal bacteria inhabiting both animals and humans are considered good indicators to monitor antimicrobial resistance as they are subjected to the continuous selection pressure of the antimicrobials [4]. The European Union (EU) and the European Food Safety Authority (EFSA) have provided guidelines for the harmonised monitoring and reporting of resistance of indicator *E. coli* and *Enterococcus* spp. [5]. Since 2014, the monitoring and reporting of the resistance of commensal *E. coli* is mandatory according to the EU decision (2013/652/EU).

In order to reduce antimicrobial resistance and give appropriate suggestions for the use of antibacterials, the survey of the resistance situation in certain regions is inevitable [5]. Setting out the current situation at a certain time point enables us to monitor changes and take appropriate measures to diminish the development of antimicrobial resistance. Similar data from different countries enable us to compare resistance of indicator bacteria and to consider possible transmission of resistant strains between countries.

The objective of this study was to estimate the occurrence of antimicrobial resistance of *E. coli* and *Enterococcus* spp. isolated from swine and cattle in Estonia from 2010 to 2015 and to study whether antimicrobial resistance differs between swine and cattle isolates.

## Methods

### Collection of study material

Faecal samples from healthy cattle and swine were collected in the course of the annual national salmonella surveillance programme carried out in Estonia in 2010–2015. According to the number of faecal samples sent to the laboratory from one herd, one to three randomly chosen samples were cultivated for the isolation of *E. coli*, *Enterococcus faecium* and *Enterococcus faecalis* as follows: one sample was selected when the total number of samples from one farm was up to 15, two samples when the sample numbers ranged between 15 and 30 samples from one farm and three samples when the number of samples from one farm varied between 31 and 50. In total, 120 *E. coli* isolates from swine and 171 *E. coli* isolates from cattle, 60 *Enterococcus* spp. isolates from cattle and 51 from swine were included in the study. The isolates originated from 38 swine (total 217 in Estonia) and 42 dairy farms (total 448 in Estonia).

*Escherichia coli* isolates (n = 206) from clinical material (post mortem samples, organ materials) originated from diseased cattle (n = 63) and swine (n = 143). These samples were sent to the National Veterinary and Food Laboratory (VFL; Tartu, Estonia) by veterinarians in 2010–2015 and all isolates were included in the study.

### Identification of *E. coli*, *E. faecium* and *E. faecalis*

The isolation and identification of *E. coli* and enterococci were performed according to accredited methods at the VFL.

For the identification of *E. coli,* the colonies were inoculated to eosin methylene blue (EMB) agar. Based on the occurrence of a green-metallic sheen that appears on the surface of the bacterial colonies after incubation at 37 °C overnight, *E. coli* was confirmed by biochemical tests (IMViC—indole, methyl red, Voges–Proskauer, Simmons citrate).

For the isolation of enterococci, 1 g of faeces was incubated at 37 °C overnight in enrichment broth (6.5% NaCl Brain Heart Infusion (BHI)), and 10 μL of enrichment suspension was spread on Slanetz-Bartley agar and incubated for 48 h at 42 °C. Up to four colonies with morphology typical of *E. faecalis*/*E. faecium* were sub-cultivated on sheep blood agar. Colonies were identified by the following criteria: haemolysis on blood agar, aesculin hydrolysis on Edwards medium, growth in presence of tellurite and the ability to ferment mannitol, sorbitol, arabinose and raffinose All pure isolates of *E. coli*, *E. faecium* and *E. faecalis* were stored (− 80 °C) for the antimicrobial susceptibility testing.

All clinical *E. coli* isolates were serotyped using *E. coli* OK O antisera for live culture produced in rabbits and F4, F5 antisera according to the manufacturer’s protocol (SSI Diagnostica A/S, Copenhagen, Denmark).

### Determination of antimicrobial susceptibility

The in vitro antimicrobial susceptibility was determined using the microdilution method (VetMIC^®^, Sweden). The susceptibility of *E. coli* isolates was tested for ampicillin, cephotaxime, nalidixic acid, chloramphenicol, florfenicol, tetracycline, colistin, gentamycin, kanamycin, streptomycin, ciprofloxacin, trimethoprim and sulphamethoxazole. The susceptibility of *E. faecalis* and *E. faecium* was tested for ampicillin, erythromycin, virginiamycin, gentamycin, streptomycin, kanamycin, tetracycline chloramphenicol, vancomycin, narasin, bacitracin and linezolid. Ampicillin was used as a test substance, whereas ampicillin covers both antimicrobial resistance ampicillin and amoxicillin.

For the interpretation of minimum inhibitory concentration (MICs) from the susceptibility testing of *Escherichia coli, E. faecalis* and *E. faecium* cut-off values available in Swedres-Svarm 2015 report Table 7.12 [6] were used.

An *E. coli* isolate was classified as multidrug-resistant (MDR) [7] when it was resistant to three or more of the following antimicrobials: ampicillin, tetracycline, chloramphenicol, colistin and florfenicol or to the following antimicrobial classes: trimethoprim/sulfamethoxazole, fluoroquinolones (ciprofloxacin or nalidixic acid), aminoglycosides (gentamicin, streptomycin or kanamycin), extended-spectrum cephalosporins (cephotaxime or cephtazidime). An *E. faecium* or *E. faecalis* was classified as MDR if the resistance was detected to any antibiotic in three or more of the following antimicrobials/antimicrobial classes: ampicillin, tetracycline, erytromycin, vancomycin, virginiamycin, aminoglycosides (gentamicin, streptomycin or kanamycin), narasin, bacitracin and linezolid.

For *E. coli* isolates resistant to either cefotaxime or ceftazidime, the phenotypic confirmatory test (National Veterinary Institute, Technical University of Denmark (DTU) scheme) for the production of ESBLs and AmpC was performed (CLSI M100-S21) [8]. Genotypic confirmation of ESBL and AmpC-positive *E. coli* (n = 16) was performed in the EU Reference Laboratory for antimicrobial resistance (EURL-AR) at DTU, where the presence of genes encoding *bla*_TEM_, *bla*_CTX_ and *bla*_SHV_ were examined. PCR assay and sequence analysis was performed at DTU as described by Xia et al. [9].

### Statistical analysis

This study material was very heterogenous, originated from clinically healthy animal and clinical submission and collected from different farms during 2010–2015. To minimize this heterogenicity, three different databases were created as follows: antimicrobial resistance of *E. coli* originated from clinically healthy animal, resistance of *E. coli* from clinical submission and resistance of *Enterococcus* spp. from healthy animal. Percentages of resistant isolates with 95% confidence intervals (95% CI) to all antimicrobial agents in both animal species (cattle and swine) were calculated. Logistic regression analysis was performed for each antimicrobial agent separately, and association between the occurrence of antibiotic resistance (0—susceptible; 1—resistant) of *E. coli* and animal species (dairy cattle *vs* swine) was studied. Due to a small number of samples, the resistance of *E. faecium* and *E. faecalis* originating from healthy animals was analysed together. Odds ratios (ORs) with 95% CIs were calculated. Similarly, the associations between multidrug-resistance (simultaneous resistance to more than three antimicrobials or antimicrobial classes) of *E. coli* from a clinically healthy animal or clinical submission and animal species were studied with logistic regression analysis. As the datasets were very unbalanced with variable number of observations from different farms and years, we fitted also logistic models considering random effects of farm and year. However, as several models corresponding to the less resistant isolates did not converge and the results of the other models were similar to the simple logistic regression analysis (including magnitude of the odds ratios and statistical significance of differences), we presented only the results of simple models. Statistical significance was assumed at ≤ 0.05. Stata 14.0 (StataCorp, Texas, USA) and SAS 9.4 (SAS Institute Inc., Cary, NC, USA) software were used for statistical analyses.

## Results

### Resistance profile of *E. coli* in healthy animals

Among the *E. coli* isolates from swine (n = 120), we found high occurrence of resistance to streptomycin (39.2%), tetracycline (32.5%) and sulfamethoxazole (30.0%). In clinically healthy cattle (n = 171), the most prevalent resistance was observed against aminoglycosides (7.0–8.8%) and tetracycline (7.0%) (Table 1).

**Table 1:** Resistance of *Escherichia coli* isolates originating from faecal samples of healthy cattle and swine and clinical submissions collected from 2010 to 2015 in Estonia

Antimicrobial	Cut-off values for resistance (mg/L)*	Healthy animals				Diagnostic submissions			
		Dairy cattle (n = 171)		Swine (n = 120)		Dairy cattle (n = 63)		Swine (n = 143)	
		%	(95% CI)	%	(95% CI)	%	95% CI	%	95%
Ampicillin^*H^	> 8	*3.5*	*(0.8 to 6.3)*	*21.5*	*(14.3 to 29.1)*	58.7	(46.5 to 70.9)	53.9	(45.7 to 62.1)
Cephotaxime	> 0.5	1.2	(− 0.4 to 2.8)	2.5	(− 0.3 to 5.3)	7.9	(1.2 to 14.6)	4.2	(0.9 to 7.5)
Cephazidime	> 0.5	2.9	(0.4 to 5.4)	3.3	(0.1 to 6.5)	7.9	(1.2 to 14.6)	7.7	(3.3 to 12.1)
Streptomycin^*H^	> 16	*7.0*	*(3.2 to 10.8)*	*39.2*	*(30.5 to 40.8)*	63.5	(51.6 to 6.4)	54.6	(46.4 to 62.8)
Gentamycin^*D^	> 4	7.0	(3.2 to 10.8)	12.5	(6.6 to 18.4)	*20.6*	*(10.6 to 30.6)*	*5.6*	*(1.8 to 9.4)*
Kanamycin	> 16	8.8	(4.6 to 13.1)	10.0	(4.6 to 15.4)	0.0	NA	0.0	NA
Ciprofloxacin^*H^	> 0.06	*0.6*	*(*− *0.6 to 1.8)*	*5.8*	*(1.6 to 10.0)*	38.1	(26.1 to 50.1)	32.2	(24.5 to 39.9)
Nalidixic acid^*D^	> 16	0.6	(− 0.6 to 1.8)	3.3	(0.1 to 6.5)	*17.5*	*(8.1 to 26.9)*	*32.2*	*(24.5 to 39.9)*
Tetracycline^*H^	> 8	*7.0*	*(3.2 to 10.8)*	*32.5*	*(24.1 to 40.9)*	58.5	(46.3 to 70.7)	60.2	(52.2 to 68.3)
Colistin^*H^	> 2	*2.4*	*(0.1 to 4.7)*	*11.6*	*(5.9 to 17.3)*	3.2	(− 1.6 to 7.6)	5.6	(1.8 to 9.4)
Chloramphenicol	> 16	2.4	(0.1 to 4.7)	5.8	(1.6 to 10.0)	9.5	(2.3 to 16.7)	18.2	(11.9 to 24.5)
Florfenicol	> 16	0.0	NA	0.8	(− 0.8 to 2.4)	0.0	NA	0.7	(− 0.7 to 2.1)
Trimethoprim^*H^	> 2	*3.5*	*(0.8 to 6.3)*	*22.4*	*(14.9 to 29.9)*	55.6	(43.3 to 67.9)	53.9	(45.7 to 62.1)
Sulfamethoxazole^*H^	> 64	*4.7*	*(1.5 to 7.9)*	*30.0*	*(21.8 to 38.2)*	60.3	(48.2 to 70.4)	68.5	(60.1 to 76.1)

* Swedres-Svarm 2015. Consumption of antibiotics and occurrence of antibiotic resistance in Sweden. Solna/Uppsala ISSN 1650-6332, 117, Table 2.17

^*H^ and ^*D^ Statistically significant difference (P < 0.05) between healthy dairy cattle and swine, and between dairy cattle’s and swine’s clinical submissions. Corresponding percentages are also presented in italic face

The resistance of *E. coli* originated from faecal samples from clinically healthy swine compared to cattle was significantly higher to ampicillin (OR = 6.5; 95% CI 2.70–15.56; P < 0.001), streptomycin (OR = 8.5, 95% CI 4.27–17.03; P < 0.001), ciprofloxacin (OR = 10.5; 95% CI 1.27–86.76; P = 0.029), tetracycline (OR = 6.4; 95% CI 3.16–12.89; P < 0.001) colistin (OR = 5.5; 95% CI 1.7–17.3; P = 0.004), sulfamethoxazole (OR = 8.7; 95% CI 3.87–19.70, P < 0.001) and trimethoprim (OR = 8.4; 95% CI 3.33–21.04; P < 0.001).

### Resistance profile of *E. coli* from diagnostic submissions

In the 143 *E. coli* isolates from swine, 136 originated from post-mortem organ material and seven isolates from animals with diarrhoea. Among the 83 *E. coli* isolates 15 different serotypes were determined. Serotyping did not show results among the rest of 60 *E. coli* isolates. The most common serotype was K88 (n = 38), followed by O138 (n = 14) and O149 (n = 12).

Out of the 63 *E. coli* isolates from dairy cattle, 18 originated from calves with signs of diarrhoea, and 45 were post-mortem samples. Among the 63 *E. coli* isolates from cattle, serotypes were confirmed in 22 isolates, where the most frequent serotype was O26.

*Escherichia coli* isolates from clinical submission showed the most prevalent resistance against sulfamethoxazole (68.6%), tetracycline (60.2%), streptomycin (54.6%), ampicillin (53.9%) and trimethoprim (53.9%). *E. coli* isolates from cattle clinical submissions were also mainly resistant to streptomycin (63.5%), sulfamethoxazole (60.3%), tetracycline (58.8%), ampicillin (58.7%) and trimethoprim (55.6%) (Table 1).

The resistance against gentamycin was significantly lower (OR = 0.17; 95% CI 0.06–0.47; P < 0.001) and resistance against nalidixic acid significantly higher (OR = 2.24; 95% CI 1.07–4.72; P = 0.034) in swine *E. coli* isolates compare to cattle isolates.

### Multidrug-resistance of *E. coli* isolates

The distribution of susceptible and MDR *E. coli* isolates from swine and cattle have shown in Table 2. The *E. coli* isolates from clinically healthy swine (n = 35; 29.2%) showed significantly higher multidrug resistance (OR = 11.2; 95% CI 4.23–29.22; P < 0.001) than the isolates from cattle (n = 6, 3.5%). The proportion of MDR isolates from clinical submission was very high both in cattle (n = 42; 66.7%) and swine (n = 93; 65.0%), without statistical differences.

**Table 2:** Distribution of susceptible and multi-drug resistant *Escherichia coli* isolates in dairy cattle and swine

Number of antimicrobials	Clinically healthy animals		Diagnostic submissions	
	Dairy cattle (n = 171)	Swine (n = 120)	Dairy cattle (n = 63)	Swine (n = 143)
Number and proportion (%) of susceptible isolates				
Susceptible to all tested anti-microbials/antimicrobials classes*	135 (78.9)	40 (33.3)	12 (19.0)	19 (13.3)
Resistant to 1–2 antimicrobials/antimicrobial classes	29 (16.9)	46 (38.3)	9 (14.3)	31 (21.7)
Number and proportion (%) of multi-drug resistant isolates				
Resistant to 3–5 antimicrobials	6 (3.5)	33 (27.5)	40 (63.5)	85 (59.4)
Resistant to 6–8 antimicrobials	0	2 (1.7)	2 (3.2)	8 (5.6)

* Antimicrobial classes: Quinolones (ciprofloxacin and nalidix acid); Aminoglycosides (streptomycin, kanamycin, gentamycin); 3th–4th generation cephalosporines (cephotaxime + cefazidime), sulfamethoxazole + trimethoprim

### Determination of ESBL- and AmpC-producing *E. coli*

All 16 *E. coli* isolates with cefotaxime and/or ceftazidime MIC above cut-off level were analysed for confirmation of ESBL and AmpC production. ESBL phenotype was confirmed in one *E. coli* isolate from clinically healthy cattle and in eight isolates from organ materials both from cattle and swine. Three *E. coli* strains out of nine exhibiting an ESBL phenotype was found to be the same genotype *bla*_TEM-52C_. All these strains originated from swine organ material that was collected post mortem.

In total, four strains representing AmpC phenotypes were found. One plasmid-encoded AmpC type β-lactamases producing *E. coli* from clinically healthy cattle was found to harbour the *bla*_CMY-1_ gene, and another from clinically healthy swine carried the *bla*_CMY-2_ gene.

### Resistance profile of enterococci

Resistance of *E. faecalis* and *E. faecium* is presented in Table 3. Altogether 51 isolates from healthy cattle and 60 isolates from healthy swine were analysed.

**Table 3:** Proportion of resistance of *Enterococcus faecalis* and *Enterococcus faecium* isolates originating from faecal samples of healthy cattle and swine in 2010–2015 in Estonia

Antimicrobial	Cut-off values for resistance (mg/L)*	Healthy animals			
		Dairy cattle (n = 51)		Swine (n = 60)	
		%	(95% CI)	%	(95% CI)
Ampicillin	> 4	0.0	NA	1.7	(− 1.6 to 5.0)
Erythromycin	> 4	21.6	(10.3 to 21.9)	26.7	(15.5 to 37.9)
Virginiamycin					
*E. faecalis*	> 32	1.9	(− 1.9 to 5.7)	5.0	(− 0.5 to 10.5)
*E. faecium*	> 4				
Gentamycin	> 32	1.9	(− 1.9 to 5.7)	1.7	(− 1.6 to 5.0)
Streptomycin**					
*E. faecalis*	> 512	*11.7*	*(2.9 to 20.5)*	*35.0*	*(22.9 to 47.1)*
*E. faecium*	> 128				
Kanamycin**	> 1024	*3.9*	*(− 1.4 to 9.2)*	*26.7*	*(1.5 to 37.9)*
Tetracycline	> 4	33.3	(20.4 to 46.2)	40.4	(27.6 to 52.4)
Chloramphenicol	> 32	1.9	(− 1.9 to 5.7)	6.7	(0.8 to 13.3)
Vancomycin	> 4	5.9	(− 0.63 to 9.4)	10.0	(2.4 to 17.6)
Narasin	> 2	3.9	(− 1.4 to 9.2)	3.3	(− 1.2 to 7.8)
Bacitracin	> 32	3.9	(− 1.4 to 9.2)	6.6	(0.4 to 13.3)
Linezolid	> 4	0.0	NA	1.7	(− 1.6 to 5.0)

* Swedres-Svarm 2015. Consumption of antibiotics and occurrence of antibiotic resistance in Sweden. Solna/Uppsala ISSN 1650-6332, 117, Table 2.17

** Statistically significant difference (P < 0.05) between resistant *Enterococcus* spp. isolates from healthy dairy cattle and swine. Corresponding percentages are also presented in italics face

Enterococci from both animal species were mainly resistant to tetracycline (33.3% in cattle, 40.4% in swine) and erythromycin (21.6% in cattle, 26.7% in swine). Enterococci from swine were also resistant to streptomycin (30.0%) and kanamycin (26.7%). Enterococci isolated from swine had a significantly higher resistance against streptomycin (OR = 4.0; 95% CI 1.46–11.14; P = 0.008) and kanamycin (OR = 8.9; 95% CI 1.91–41.66; P = 0.006) compared to isolates from cattle. The proportion of fully susceptible *Enterococcus* spp. isolates was 49% (n = 25) in cattle and 35% (n = 21) in swine. Multidrug resistance was significantly higher (OR = 4.4; 95% CI 1.17–16.78; P = 0.029) in swine isolates (n = 13) than in isolates that originated from cattle (n = 3).

## Discussion

This study is the first broad-based overview of antimicrobial resistance of these animal pathogens in Estonia. Currently, there is an extensive movement of live animals and food of animal origin between countries and continents. Regarding the possible transfer of resistant microbes, overview of the situation in each region cannot be underestimated [10].

In our study, the proportion of resistant *E. coli* isolates and MDR *E. coli* isolates originating from healthy swine was higher than that of *E. coli* isolates that originated from healthy cattle. The resistance against tetracycline, ampicillin, streptomycin, sulfamethoxazole, trimethoprim, ciprofloxacin and colistin differed significantly. Monitoring programmes from Finland, the Netherlands and Denmark have also described higher resistance among the swine isolates [11–13].

Isolates originating from swine were more resistant to mainly orally administered antibiotics. For instance, doxycycline, ampicillin/amoxicillin and sulpha/trimethoprim have been used for the treatment of swine diseases in a large volume and over a long time period in Estonia [10], (unpublished data from the Estonian State Agency of Medicines). There are about 86,900 dairy cows (in total 448 farms) and 298,000 pigs (in total 217 farms) in Estonia. In 2012–2016 the average amount of tetracycline used for the treatment of swine was about 1500 kg of pure active substance per year, for the treatment of cattle about 210 kg/year (data from the Estonian State Agency of Medicines). The same figures for ampicillin/amoxicillin were 2200/500 and for sulpha/trimethoprim 110/50, respectively. In Estonia, tetracyclines (including doxycycline), ampicillin/amoxicillin and sulpha/trimethoprim are authorised for oral treatment in swine and poultry, not in cattle (data from the Estonian State Agency of Medicines). Considering this we can say that in Estonia, there might be a link between the use of antibiotics and the level of resistance, and enteric bacteria in pigs are more often exposed to antibiotics than in cattle. There is a higher probability for commensal *E. coli* to become a reservoir of resistance when oral antibiotics are widely used in the swine farms. Several authors have confirmed that oral administration of antibiotics to pigs increases the level of antimicrobial resistance [14, 15] and there is a strong correlation between the use of antimicrobials and the extent of antimicrobial resistance in *E. coli* isolated from livestock [16, 17]. This could explain the high resistance of commensal *E. coli* strains isolated from healthy swine in our study, as oral antibiotics are not commonly used for the treatment of cattle in Estonia.

We found high resistance to ciprofloxacin and nalidixic acid in bacteria originating from diseased animals in both animal species. Enrofloxacin and other quinolones are still used quite extensively for the treatment of swine and cattle in Estonia (amounts of active ingredients 85/55 kg per year respectively (unpublished data from the Estonian State Agency of Medicines)). It is not in line with the local rules of prudent use of antimicrobials [18]. That could explain the high resistance to quinolones as there can be a link between the presence of antibiotics in the body and the number of resistant bacteria [19]. Monitoring of resistance to fluoroquinolones should be continued in future studies as well as resistance to virginiamycin and chloramphenicol—compounds which are not used in veterinary practice in Estonia and which resistance can be associated with the use of tetracyclines at low concentrations [20].

We found considerable phenotypic resistance to colistin in *E. coli* isolates from healthy swine. We did not investigate colistin genotypic resistance in this study. However, colistin resistance of swine *E. coli* should be focused in future studies as a plasmid carrying the colistin resistance gene *mcr*-*1* was isolated from a pig slurry sample in Estonia [21]. Some isolates of vancomycin resistant enterococci were isolated from healthy swine, the genotypic conformation and possible link with the use of antibiotics should be focused in future studies.

We did not analyse the difference in the resistance of *E. coli* isolates from healthy animals and diagnostic submissions because the origin and collection of that kind of material is different, and comparison may lead to biased conclusion, although higher number of resistant isolates among the clinical submissions were observed, which is in line with the results of other authors [12, 13, 22]. Isolates from clinical submission can be more frequently resistant than isolates from healthy animals because of the more frequent exposure to antimicrobials, and in veterinary practice we need to keep in mind that the use of antimicrobial agents may select bacteria carrying virulence genes [23].

Resistance of enterococci, as well as development of multidrug resistance was lower in cattle isolates compared to swine isolates, which is also reported in other investigations [11–13].

This was the first time in Estonia when the ESBL-producing *E. coli* harbouring the *bla*_TEM-52C_ genotype was found in swine post-mortem tissue samples. TEM-52 and CTX-M are often the most dominant types of enzymes in swine in other countries [24–26]. Several studies [27–30] have reported that strains producing AmpC and ESBL are often resistant to multiple agents. As faecal carriage of plasmid-mediated AmpC β-lactamases was found in healthy swine and cattle, the possible development and transmission methods of antimicrobial resistance in cattle and swine must be investigated in future studies.

## Conclusions

The highest percentages of drug resistance in isolates of *E. coli* were detected to streptomycin, tetracycline, sulfamethoxazole, trimethoprim, ampicillin and colistin.

The number of MDR *E. coli* isolates was significantly higher in clinically healthy swine compared to that in cattle. The antimicrobial resistance of *E. faecalis* and *E. faecium* to erythromycin and tetracycline was high in both animal species, in swine enterococci it was high also to streptomycin and kanamycin.

This broad-based overview of antimicrobial resistance of these animal bacteria creates a basis for the future investigations and analyses of the resistance development in Estonia. In light of this, we strongly recommend assessment of the treatment plans in the swine industry in Estonia in order to ensure the prudent use of antimicrobials and to minimise the potential spread of resistant bacteria from swine to the environment and to humans.
